# Molecular characterisation of extended-spectrum ß-lactamase producing *Escherichia coli* in wild birds and cattle, Ibadan, Nigeria

**DOI:** 10.1186/s12917-020-02734-4

**Published:** 2021-01-18

**Authors:** Kayode Fashae, Ines Engelmann, Stefan Monecke, Sascha D. Braun, Ralf Ehricht

**Affiliations:** 1 Department of Microbiology, University of Ibadan, Ibadan, Nigeria; 2 BLINK AG, Jena, Germany; 3 InfectoGnostics Research Campus, Jena, Germany; 4 Leibniz Institute of Photonic Technology (Leibniz-IPHT), Jena, Germany; 5 Institut für Medizinische Mikrobiologie und Hygiene, Medizinische Fakultaet “Carl Gustav Carus”, Technische Universitaet Dresden, Fiedlerstr 42, 01307 Dresden, Germany; 6 Friedrich Schiller University Jena, Institute of Physical Chemistry, Jena, Germany

**Keywords:** ESBL, Microarray, Africa, CTX-M, Wild birds, Cattle

## Abstract

**Background:**

Antimicrobial resistance (AMR) is an increasing global health concern reducing options for therapy of infections and also for perioperative prophylaxis. Many *Enterobacteriaceae *cannot be treated anymore with third generation cephalosporins (3GC) due to the production of certain 3GC hydrolysing enzymes (extended spectrum beta-lactamases, ESBLs). The role of animals as carriers and vectors of multi-resistant bacteria in different geographical regions is poorly understood. Therefore, we investigated the occurrence and molecular characteristics of ESBL-producing *Escherichia coli* (*E. coli*) in wild birds and slaughtered cattle in Ibadan, Nigeria.

Cattle faecal samples (*n* = 250) and wild bird pooled faecal samples (cattle egrets, *Bubulcus ibis*, *n* = 28; white-faced whistling duck, *Dendrocygna viduata, n* = 24) were collected and cultured on cefotaxime-eosin methylene blue agar. Antimicrobial susceptibility was determined by agar diffusion assays and all 3GC resistant isolates were genotypically characterised for AMR genes, virulence associated genes (VAGs) and serotypes using DNA microarray-based assays.

**Results:**

All 3GC resistant isolates were *E. coli*: cattle (*n* = 53), egrets (*n* = 87) and whistling duck (*n* = 4); cultured from 32/250 (12.8%), 26/28 (92.9%), 2/24(8.3%), cattle, egrets and whistling duck faecal samples, respectively. *bla*CTX-M gene family was prevalent; *bla*CTX-M15 (83.3%) predominated over *bla*CTX-M9 (11.8%). All were susceptible to carbapenems. The majority of isolates were resistant to at least one of the other tested antimicrobials; multidrug resistance was highest in the isolates recovered from egrets.

The isolates harboured diverse repositories of other AMR genes (including *strB* and *sul2*), integrons (predominantly class 1) and VAGs. The isolates recovered from egrets harboured more AMR genes; eight were unique to these isolates including *tetG*, *gepA*, and *floR*. The prevalent VAGs included *hemL* and *iss*; while 14 (including *sepA*) were unique to certain animal isolates*. E. coli* serotypes O9:H9, O9:H30 and O9:H4 predominated. An identical phenotypic microarray profile was detected in three isolates from egrets and cattle, indicative of a clonal relationship amongst these isolates.

**Conclusion:**

Wild birds and cattle harbour diverse ESBL-producing *E. coli* populations with potential of inter-species dissemination and virulence. Recommended guidelines to balance public health and habitat conservation should be implemented with continuous surveillance.

## Background

Antimicrobial resistance (AMR) in bacteria is an increasing and widespread public health concern which has grossly eroded the efficacy of many antimicrobial substances. Multidrug resistant – and especially beta-lactam-resistant – bacteria have further limited therapeutic options, leading to treatment failure [1], prolonged stay of patients in hospitals and the need for other and usually more expensive and/or more toxic drugs [2, 3]. Of particular concern are extended-spectrum β-lactamase (ESBL)-producing bacteria which are resistant to third generation cephalosporins (3GC) through hydrolysis of these antimicrobials [4, 5] and which pose especially serious treatment challenges [6]. ESBL genes could spread through clonal dissemination of host bacteria or via mobile genetic elements [7, 8]. ESBL genes of the CTX-M family are the most common with *bla*CTX-M-1/15 being most prevalent [9]. The prevalence of different types of CTX-M enzymes also varies with geographical areas [10]. The family *Enterobacteriaceae*, particularly *E. coli*, are common ESBL-producers. They inhabit animal and human guts and could cause diverse infections [8, 11].

While AMR is a global phenomenon [12], the situation is worsened in the developing countries including Nigeria by lack of antimicrobial regulation policies, unrestricted access and indiscriminate use of antimicrobials, and the lack of national antimicrobial resistance surveillance programmes.

AMR is a complex public health challenge involving humans, animals (wildlife, livestock and companion animals) and the environment [13–18]. Exchange of antimicrobial resistance genes and/or host bacteria can occur between animals and humans; thus, warranting a unified-health approach to its control. Food animals including cattle are reservoirs of ESBL-producing *Enterobacteriaceae* that could disseminate through the food chain and/or animal contact [8, 19, 20]. Reports on ESBL-producing *Enterobacteriaceae* in food animals in Africa including Nigeria are scarce [13, 15, 21]. *E. coli* can be grouped into commensals and pathogenic strains based on the presence of some virulence-associated genes (VAGs); the latter are further divided into pathotypes, harbouring different sets of VAGs and are associated with different infections [22, 23].

The role of wild animals, particular wild birds, as reservoirs and vectors of antimicrobial resistant bacteria is increasingly appreciated. ESBL-producing *Enterobacteriaceae* have been reported in at least 80 species of wildlife dominated by wild birds, with the most prevalent species being *E. coli* [18]. However, there are only few reports from Africa [24]. Wild animals are not directly exposed to antibiotics but rather indirectly, through environmental contamination. They could be infected or colonised by resistant bacteria through feeding on contaminated animal wastes or carcasses and drinking of contaminated water. In Nigeria, egrets are common in the South-Western regions (especially during the dry season), and they are often associated with cattle herds, feeding on the cattle ticks and scavenging the faeces. They are also scavengers in urban areas, particularly in abattoirs.

This study determined the occurrence of ESBL-producing *Enterobacteriaceae* in two populations of wild birds (egrets and whistling ducks) and cattle at slaughter. The genes encoding ESBLs and other antimicrobial resistance genes as well as virulence-associated genes were determined. The serotypes of all *E. coli* isolates were determined by serogenotyping (https://onlinelibrary.wiley.com/doi/full/10.1111/1348-0421.12120).

## Results

A majority (26/28, 92.9%) of the collected egret faecal samples yielded bacterial growth (bacterial colonies) on cefotaxime-eosin methylene blue agar (CEMB) plates; while 32/250 (12.8%) and 2/24 (8.3%) of cattle and whistling duck faecal samples yielded growth, respectively.

From the CEMB plates, 88 (egret), 4 (duck) and 55 (cattle) putative *E. coli* isolates were picked for further analysis; in addition to one unassigned bacterial isolate each from egret and cattle faecal samples. All the putative *E. coli* isolates were identified by microarray using the *E. coli* PanType Kit. The unassigned bacterial isolate from the egrets was identified as *Citrobacter freundii* and the one from cattle as *Salmonella enterica spp. enterica* serovar Tees (Table 1).

**Table 1:** Overview of all collected animal faecal samples and number of yielded bacterial isolates

Animals	Collected faecal samples	No. samples yielding bacterial growth on CEMB^**d**^	Total bacterial isolates	***E. coli*** isolates	Non-***E. coli*** isolates	Cefotaxime resistant ***E. coli*** isolates^**a**^	Cefotaxime susceptible ***E. coli*** isolates	Non-***E. coli*** isolates resistant to cefotaxime
Cattle	250	32	56	55	1^b^	53	2	0
Egret	28	26	89	88	1^c^	87	1	0
Whistling duck	24	2	4	4	0	4	0	0
Total	302	60	149	147	2	144	3	0

^a^ Subjected to further analyses (antimicrobial susceptibility testing to other antimicrobials; microarray-based analysis of antimicrobial and virulence genes and serotypes)

^b^ *Salmonella enterica* spp. *Enterica* serovar Tees

^c^ *Citrobacter freundii;* ^d^*CEMB (cefotaxime-eosine methylene blue agar plates)*

A total of 87/88 (98.9%) (egret), 4/4 (100%) (whistling duck) and 53/55 (96.4%) (cattle) *E. coli* isolates exhibited cefotaxime resistance (Tables 1 and 2). The *Salmonella* and *Citrobacter* isolates were susceptible to cefotaxime (Table 1).

**Table 2:** Distribution of cefotaxime resistant E. coli (CREC) isolates cultured from faecal samples of cattle and wild birds^a, b, c^

Sample codes	No. CREC^**d**^	Sample codes	No. CREC	Sample codes	No. CREC	Sample codes	No. CREC
cattle 10	1	cattle 136	1	cattle 238	2	egrets 4.6	4
cattle 15	1	cattle 138	2	cattle 240	1	egrets 4.7	2
cattle 29	2	cattle 139	1	egrets 1.1	1	egrets 4.8	4
cattle 70	2	cattle 144	2	egrets 2.1	5	egrets 4.9	1
cattle 82	1	cattle 146	2	egrets 2.3	5	egrets 4.10	5
cattle 87	2	cattle 151	2	egrets 2.4	4	egrets 4.11	4
cattle 101	2	cattle 152	2	egrets 2.5	5	egrets 4.12	4
cattle 104	1	cattle 170	2	egrets 2.6	3	egrets 5.1	2
cattle 109	1	cattle 175	1	egrets 2.7	4	egrets 5.2	3
cattle 115	2	cattle 180	2	egrets 2.8	1	egrets 5.3	4
cattle 120	2	cattle 182	2	egrets 4.1	5	egrets 5.4	2
cattle 124	2	cattle 204	3	egrets 4.2	5	egrets 5.5	4
cattle 127	2	cattle 223	1	egrets 4.3	3	egret 5.6	2
cattle 131	2	cattle 231	2	egrets 4.4	2	whisling duck 13	3
cattle 135	1	cattle 235	1	egrets 4.5	3	whistling duck 21	1

^a^ For egrets and whistling ducks, each sample is a pool of five different faecal samples collected on the field

^b^ 218/250 (cattle), 2/28 (egrets) and 22/24 (whistling duck) faecal samples that yielded no cefotaxime resistant isolates are not shown

^c^ Some faecal samples yielded more than one CREC resistant *E. coli* isolate

^d^ Number of *E. coli* isolates found resistant to cefotaxime after antimicrobial susceptibility testing of multiple bacterial colonies cultured from each sample on cefotaxime eosine methylene blue agar

All the cefotaxime resistant *E. coli* (CREC) isolates from the three animal populations with the exception of three egret isolates (LEK 22, LEK 23 and LEK 28) and one cattle isolate (Ct 9) also revealed resistance to at least one other tested antimicrobial (Table 3).

**Table 3:** Antimicrobial resistance patterns of the cefotaxime-resistant *E. coli* isolates

R-Type^**a**^	Egret (***n*** = 87)	Duck (***n*** = 4)	Cattle (***n***= 53)
CTX	3 (3.4)	0 (0.0)	1 (1.9)
CTX, CP	1 (0.1)	0 (0.0)	0 (0.0)
CTX, CP, STR, CHL	0	0 (0.0)	1 (1.9)
CTX, CP, TET	2 (2.3)	0 (0.0)	3 (5.7)
CTX, CP, TET, CHL	1 (0.1)	0 (0.0)	1 (1.9)
CTX, CP, TET, CHL, STR	2 (2.3)	0 (0.0)	0 (0.0)
CTX, CP, TET, GEN	6 (6.9)	0 (0.0)	0 (0.0)
CTX, CP, TET, GEN, CHL	0 (0.0)	0 (0.0)	2 (3.8)
CTX, CP, TET, GEN, CHL, STR	19 (21.8)	0 (0.0)	0 (0.0)
CTX, CP, TET, GEN, STR	3 (3.4)	1 (25.0)	0 (0.0)
CTX, CP, TET, STR	4 (4.6)	0 (0.0)	0 (0.0)
CTX, CP, TET, STR, GEN	0 (0.0)	0 (0.0)	2 (3.8)
CTX, CP, TET, STR, GEN, CHL	0 (0.0)	0 (0.0)	9 (17.0)
CTX, GEN, CHL	1 (0.1)	0 (0.0)	0 (0.0)
CTX, STR	0 (0.0)	3 (75.0)	4 (7.5)
CTX, TET	7 (0.8)	0 (0.0)	2 (3.8)
CTX, TET, GEN, STR	2 (2.3)	0 (0.0)	0 (0.0)
CTX, TET, STR	36 (41.4)	0 (0.0)	28 (52.8)

^a^*CTX* cefotaxime, *CP* ciprofloxacin, *TET* tetracycline, *GEN* gentamicin, *CHL* chloramphenicol, *STR* streptomycin

The egret CREC isolates showed highest resistance rate to tetracycline (85/87) followed by streptomycin (69/87) and ciprofloxacin (38/87) (Table 4). The duck CREC isolates showed the highest resistance rate against streptomycin (4/4) followed by equal resistance rate to each of ciprofloxacin, tetracycline and gentamicin (1/4) (Table 4).

**Table 4:** Antimicrobial resistance of cefotaxime-resistant *E. coli* (CREC) isolates of the animals to other antimicrobials

	No. (%) Resistance		
Antimicrobials	Egrets (***n*** = 87)	Ducks (***n*** = 4)	Cattle (***n*** = 53)
Gentamicin	32 (36.7)	1 (25.0)	13 (24.5)
Streptomycin	69 (79.3)	4 (100)	46 (86.8)
Ciprofloxacin	38 (43.7)	1 (25.0)	17 (32.1)
Tetracycline	85 (97.7)	1 (25.0)	47 (88.7)
Chloramphenicol	22 (25.3)	0 (0.0)	13 (24.5)

For the cattle CREC isolates, tetracycline resistance (47/53) was the most commonly detected phenotype. Streptomycin (46/53) and ciprofloxacin resistance (17/53) was also common (Table 4). Multidrug resistance (MDR, resistance to three or more antimicrobial classes) was also observed in many of the CREC isolates: egrets (n = 76), duck (n = 1) and cattle (n = 46) (Table 3). The MDR to cefotaxime, tetracycline and streptomycin (R-type: CTX, TET, STR) predominated amongst both egrets (36/87; 41.4%) and cattle (28/53; 52.8%) isolates. MDR to cefotaxime, ciprofloxacin, tetracycline, gentamicin and streptomycin (CTX, CP, TET, GEN, STR) was found in a single duck isolate (Table 3). All the CREC isolates were susceptible to tested carbapenems.

**Fig. 1:**
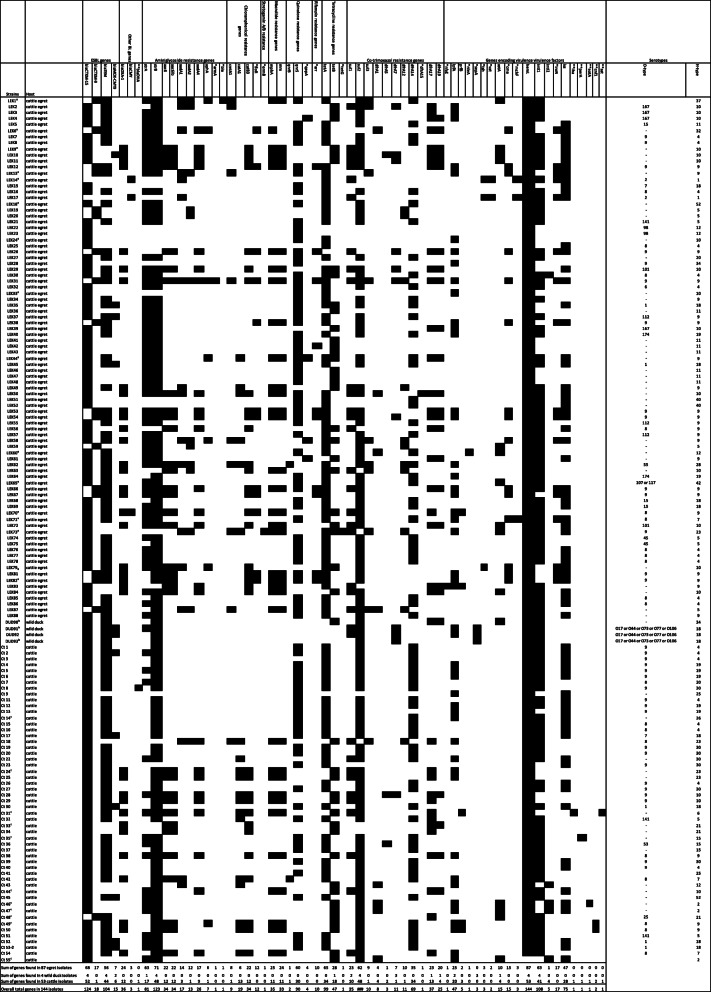
Overview of detected antimicrobial resistance and virulence genes in and serotypes of wild birds and cattle ESBL-producing *E. coli* isolates. *Not detected in the cattle ESBL-producing *E. coli* isolates; ** Not detected in the wild bird ESBL-producing *E. coli* isolates. Black boxes indicate gene detection; minus sign under serotype column indicates not detected; a) Egret (*Bubulcus ibis*) CREC isolates representing the 16 different detected virulence genotypes; b) whistling duck (*Dendrocygna viduata*) CREC isolates representing the 3 different detected virulence genotypes; c) Cattle CREC isolates representing the 11 different detected virulence genotypes

All the CREC isolates were ESBL-producers; they all harboured ESBL gene *bla*CTX-M1/15 or *bla*CTX-M9 with the exception of two egret CREC isolates (LEK 14 and LEK 17). The *bla*CTX-M1/15 gene was more prevalent than the *bla*CTX-M9 gene among the CREC isolates: egret, 68 (78.2%) vs. 17 (19.5%); duck, 4 (100%) vs. 0 (0%); and cattle, 52 (98.1%) vs. 1 (1.9%) (Fig. 1), respectively. None of the isolates contained both *bla*CTX-M1/15 and *bla*CTX-M9 genes. The *bla*CTX-M genes were not detected by the microarray-based assays in two cattle egret CREC isolates LEK 14 and LEK 17 but these isolates both harboured the *bla*CMY gene (Fig. 1). One egret isolate (LEK 70) harboured both *bla*CTX-M1/15 and *bla*CMY (Fig. 1). Consensus sequences for *bla*TEM were found in 56 (64.4%), 4 (100%), 44 (83.0%) of the egret, duck and cattle isolates, respectively. Consensus sequences of the *shv* gene family were not detected in any of the isolates. The *Citrobacter* and *Salmonella* isolates did not contain any ESBL genes. There was full concordance between the cefotaxime resistance and ESBL genotype.

Several other antimicrobial resistance genes were detected in the CREC isolates. The most prevalent antimicrobial resistance gene in the egret isolates was *strB* (81.6%) followed by *tetA* (74.7%). The *strB* and *sul2* genes predominated in the duck isolates with equal prevalence rates (100%); these two genes were also highly prevalent in the cattle isolates (90.6%) (Fig. 1).

Overall, more antimicrobial resistance genes were found in the egret CREC isolates than in the cattle isolates. Eight of the detected non-beta-lactam antimicrobial resistance genes were exclusively found in the egret CREC isolates; *armA* (aminoglycoside resistance) (*n* = 1), *ble* (aminoglycoside) (*n* = 1), *tetG* (tetracycline) (*n* = 1), *ermB* (macrolide/streptogramin) (*n* = 1), *arr* (rifampicin) (*n* = 10), *dfrA19* (trimethoprim) (*n* = 20), *gepA* (fluoroquinolone) (*n* = 4) and *floR* (chloramphenicol) (*n* = 12) (Fig. 1).

Class 1 integrons were more prevalent than class 2 integrons in the CREC isolates amongst the isolates recovered from all three animal hosts: egret, 72.4% (*n* = 63) vs. 1.1% (*n* = 1); cattle, 77.4% (*n* = 41) vs. 7.5% (*n* = 4); whistling duck, 100% (*n* = 4) vs. 0% (Fig. 1).

Based on the combinations of the ten aminoglycoside genes detected in egret CREC isolates, 13 different genotypes were observed. The most prevalent (14/87) genotype harboured the *aac6*, *aac6Ib* and *aadA4* genes (Fig. 1). Eight aminoglycoside genes were found in the cattle CREC isolates. The genotype harbouring the *aac6*, *aac6Ib* and *aadA4* genes was the most prevalent. Only two aminoglycoside genes were found in the whistling duck CREC isolates (Fig. 1).

**Fig. 2:**
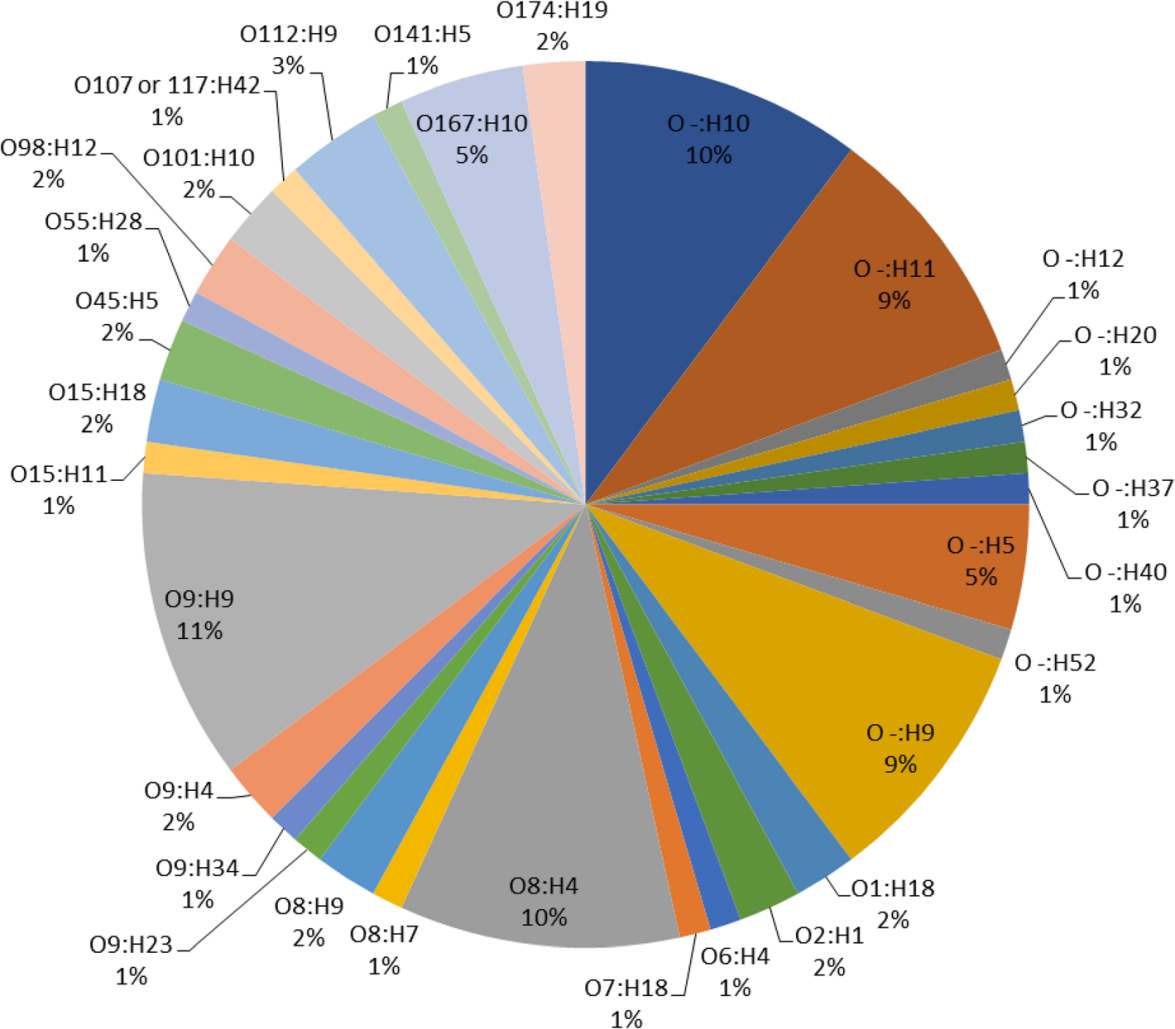
Overall distribution of serotypes of cattle egret ESBL-producing *E. coli* isolates

**Fig. 3:**
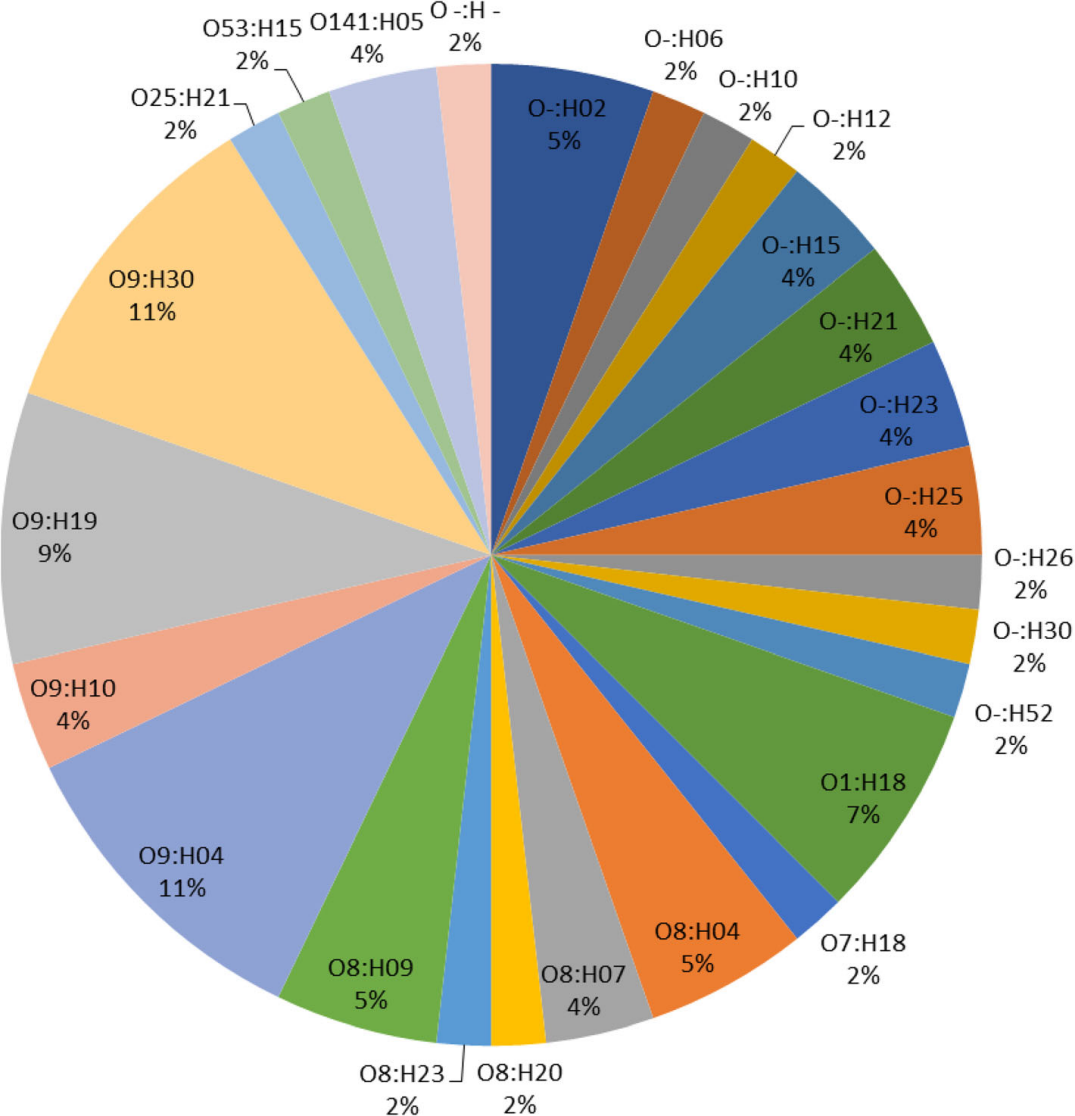
Overall distribution of serotypes of ESBL-producing *E. coli* isolates of cattle

Analysis of O- and H-types of *E. coli* isolates by SeroGenoTyping AS-1 Kit showed a high diversity of serotypes. A total of 52 (egret), three (duck) and 36 (cattle) CREC isolates could be identified as 22, one and 14 distinct serotypes, respectively (Figs. 1, 2 and 3). The remaining CREC isolates could not be assigned to serotypes as only the H-antigens could be determined. The serotype O9:H9 predominated among the egret CREC isolates (10/87) (Figs. 1, 2). The serotype O9:H30 and O9:H4 were equally prevalent among the cattle CREC isolates (6/53) (Figs. 1 and 3). While three out of the four duck CREC isolates revealed O17/O44/O73/O77/O106:H18 serotype (Fig. 1).

The O8 and O9 antigens predominated among the egrets (*n* = 26; 29.9%) and cattle (*n* = 29; 54.7%) isolates. The *E. coli* serotypes containing the O8 and O9 antigens included O8:H4, O8:H7, O8:H9, O9:H4, O9:H9, O9:H23, and O9:H34 (egrets); and O8:H20, O8:H4, O8:H23,O8:H9, O8:H7, O9:H4, O9:H19, O9:H30, and O9:H10 (cattle) (Figs. 1, 2 and 3).

Seven distinct serotypes were identified amongst the egret and cattle isolates; O8:H4; O8:H7, O8:H9, O9:H4, O1:H18, O7:H18 and O141:H5. The prevalence rates of serotypes O8:H4 and O9:H4 varied between egret and cattle isolates: serotype O8:H4 was more prevalent in egrets (10% vs. 5%) while serotype O9:H4 was more prevalent in cattle (11% vs. 2%) (Figs. 2 and 3). The duck isolates did not share any serotype with the isolates from other animals. Notably, the same serotype (O8:H4), virulence- and AMR-genotype was identified in one egret (LEK 85) and two cattle isolates (Ct 15 and Ct 16), suggesting clonality (Fig. 1).

Twelve virulence associated genes (VAGs) were detected in the egret CREC isolates. The *hemL* gene was detected in all the isolates while the other 11 VAGs had a prevalence of equal to or less than 55.2% (Fig. 1). The second most prevalent VAG was *iss* (increased serum survival) (55.2%) followed by *lpfA* (28.7%), *iroN* (19.5%), *cma* (17.2%) and *astA* (11.5%). Nine VAGs, however, were detected in the cattle CREC isolates. *hemL* was also detected in all the cattle isolates while the other VAGs were detected at a prevalence of ≤ 54.7% (Fig. 1). The second most prevalent VAG in the cattle isolates was also *iss* (54.7%) followed by *lpfA* (37.7%). Only three VAGs were found in the duck CREC isolates; *sepA* (three isolates), *lpfA* (two isolates) and *astA* (one isolate) (Fig. 1).

Eight VAGs were exclusively detected in the egret isolates: *iroN* (siderophore receptor) (17/87), *cma* (15/87), *vat* (vacuolating autotransporter toxin) (2/87), *tsh* (temperature-sensitive haemagglutinin) (3/87), *mchF* (3/84), *nleA* (1/87) and *nfaE* (non-fimbrial adhesin) (1/87) (Fig. 1). While *perA* (1/53), *eatA* (1/53), *cnf1* (cytotoxic necrotizing factor) (2/53), *sat1* (secreted autotransporter toxin) (1/53) and *iha* (adhesin-siderophore receptor) (1/53) were detected only in the cattle (Fig. 1) and *sepA* (3/4) in the duck isolates (Fig. 1).

Two VAGs were found exclusively in the CREC isolates of egret and cattle: *iss* (47/87 vs. 27/53) and *prfB* (2/87 vs. 3/53). While three VAGs (*hemL, lpfA* and *astA*) were common to the egret, cattle and whistling duck CREC isolates; only *hemL* was found in all isolates of the three animal hosts; while *lpfA and astA* had different prevalence rates in the three animal hosts. The prevalence rates of *lpfA* were: 25/87 (28.7%) (egret), 20/53 (37.7%) (cattle), 2/4 (50%) (whistling duck), while the prevalence rates of *astA* were: 10/87 (11.5%) (egret), 4/53 (7.5%) (cattle) and 1/4 (25%) (whistling duck).

A total of 16, 11 and three virulence genotypes were observed in egret, cattle and duck *E. coli* isolates, respectively (Fig. 1). The VAG genotype harbouring *hemL* and *iss* (*as* in LEK 44 and Ct 44 isolates) predominated the egret and cattle isolates; 25/87 (28.7%) (egret) and 18/53 (34.0%) (cattle), followed by genotype encoding only *hemL (as in* LEK 18 and Ct 33 isolates) with the prevalence rates of 24/87 (27.6%) (egret) and 14/53 (26.4%) (cattle). The third prevalent VAG genotype in the egrets harboured *lpfA*, *cma*, *hemL*, *iroN* and *iss* (as in LEK 13 isolate) with the prevalence rate of 10/87 (11.5%). While, the third most prevalent VAG genotype in the cattle isolates harboured *lpfA*, *hemL* and *iss* (as in Ct 14 isolate) with the prevalence rate of 9/53 (17.0%). The VAG genotype encoding *lpfA* and *hemL (*as in LEK 24 and Ct 24 isolates) was shared by the isolates of egret (10/87) (11.5%) and cattle (7/53) (13.2%). While the genotype harbouring *astA* and *hemL* (as in LEK 1, Ct 48 and DUD 90 isolates) was detected in the isolates of the three animal hosts with very low prevalence rates; egret (2/87) (2.3%), cattle (1/53) (1.9%) and whistling duck (1/4) (25%).

Varied proportions of CREC isolates of the three animal hosts harboured multiple VAGs (encoding more than 3 VAGs): 26/87 (29.9%) (egret), 14/53(26.4) (cattle) and 2/4 (50%) (egret). The majority (9/10) of the egret isolates having the VAG genotype encoding *lpfA*, *cma*, *hemL*, *iroN* and *iss* also showed multidrug resistance to antimicrobials from five antimicrobial classes (cefotaxime, ciprofloxacin, tetracycline, gentamicin, chloramphenicol and streptomycin) compared to only 1/10 isolates showing resistance to only three antimicrobials (cefotaxime, gentamicin and chloramphenicol). Furthermore, all 10 egret isolates having the VAG genotype encoding *lpfA*, *cma*, *hemL*, *iroN* and *iss* belonged to serotype O9:H9.The serotype O9:H9 was not common among egret isolates having other VAG genotypes (Fig. 1). Also, the majority (5/8) of cattle isolates having the VAG genotype encoding *lpfa*, *heml* and *iss* belonged to serotype O9:H19 compared with only (3/8) belonging to other serotypes (Fig. 1).

Overall, none of the *E. coli* isolates could be categorized as one of the molecularly recognised pathogenic strains of *E. coli* [22, 23] based on the VAGs detected.

## Discussion

Our study shows a high carriage rate of ESBL-producing *E. coli* in cattle egrets and cattle and thus these animals might constitute public health risk. The poor meat hygiene practices in Nigeria could facilitate meat contamination and dissemination during slaughter and transport could lead to a spread to humans through the food chain [25]. The common intermingling of animals with humans in the country could also promote a further spread to humans.

Antimicrobial usage is an important driver of antimicrobial resistance [12]. This factor probably plays a role in the prevalence of antimicrobial resistance in the cattle isolates considering the high and indiscriminate usage of antimicrobials in veterinary and human medicine and animal production in Nigeria [26] as in many African countries [27]. This issue is exacerbated by lack of an antimicrobial control policy and surveillance, off-counter availability of antimicrobials, sub-standard antimicrobials and employment of non-professionals who administer antimicrobials to animals. The high prevalence of MDR isolates is a public health concern as this will complicate treatment of infections caused by these bacteria due to limited therapeutic options. Since wild birds are not directly exposed to antimicrobials, they probably acquired the antimicrobial resistant bacteria through exposure to the environment contaminated with agricultural, animal and human waste.

Wild birds are environmental sentinels for the spread of antimicrobial resistance [18]. The cattle egret and whistling duck are migratory birds and could therefore further spread the bacteria along their transboundary migration routes contaminating water (including those used for irrigational, recreational and drinking purposes), agricultural land and farm produce [28]. They could act as a source of antimicrobial resistant *E. coli* and resistance determinants to other wildlife [29] through which wild animals including those hunted for meat consumption can also be contaminated. It is noteworthy that the study site for the wild birds is by the bank of a river which supplies raw water to the Water Treatment Plant supplying ‘treated water’ for domestic use of an entire University community. This puts the community at a particular risk due to possibility of contamination of the water by faecal droppings of the birds; the risk is further heightened by the apparent inadequate treatment of the water. Fresh food producing farms were also located close to the study site posing additional risk from the faecal contamination of their products [30]. Additionally, contamination of roof tops by the faecal droppings of the birds could lead to contamination of harvested rain water leading to further transmission through usage of rain water [31], which is common in this environment due to water shortage. Also concerning is the mingling of egrets with cattle at abattoirs, cattle markets and grazing fields through which the birds can spread the bacteria and/or resistance determinants to cattle with the potential of ultimately entering the food chain. A transmission of resistant bacteria from cattle to the egrets is also possible as the birds scavenge abattoir wastes. The indicated phenotypic and genotypic similarity between an egret isolate (LEK 85) and two cattle isolates (Ct 15 and Ct 16) isolates suggests transmission between egrets and cattle reflecting the association of these egrets with cattle. The general practice of slaughtering of animals in open fields in abattoirs make carcasses susceptible to faecal contamination from faecal droppings of egrets and other birds; this calls for biosafety measures in the abattoirs.

The high prevalence of ESBL-producing *E. coli* in cattle corroborates earlier reports from Africa [13]. An even higher prevalence of 46.6% was reported in Egypt from 210 rectal swab samples collected from dairy cattle [15].

The resistance of these bacteria to other antimicrobials, including ciprofloxacin, would further complicate treatment; however, all the bacteria were susceptible to carbapenems. The susceptibility to carbapenems corresponds to the general low prevalence of carbapenem resistant *E. coli* in food producing animals in Africa [13, 15, 32].

The predominance of CTX-M15 reported in this study is consistent with the findings in chickens [30] and humans (clinical and asymptomatic) in Nigeria [21, 33] and the global epidemiology of the CTX-M family, including Africa [13]. CTX-M15 is more widespread compared to other CTX-M types which are found in certain locales or host species. The study marks first report of the occurrence of CTX-M9 in Nigeria showing egret as a more prominent reservoir compared to cattle. Report of CTX-M9 in food animals (cattle and chicken) in Africa is not common [15, 34]. It is also noteworthy that *E. coli* isolates recovered from egrets harboured more antimicrobial resistance genes than those recovered from the other animal species; with exclusive detection of eight antimicrobial resistance genes; *armA* and *ble* (aminoglycoside), *tetG* (tetracycline), *ermB* (macrolides/streptogramin), *arr* (rifampicin), *dfrA19* (trimethoprim), *gepA* (fluoroquinolone), *floR* (chloramphenicol) in the egret isolates. This probably reflects the migratory and scavenging nature of the egrets. The absence of *floR* gene in the cattle isolates contrasts to the high prevalence of this gene in dairy cattle isolates recovered elsewhere in Africa, such as Egypt [15]; while a *floR* prevalence rate of 33% was reported in European cattle isolates [8]. The *floR* gene confers resistance to florfenicol (a fluorinated derivative of chloramphenicol) approved for use in cattle in Europe since 1995 [35].

A majority of the bacteria harboured ≤ 2 VAG. Based on detection of VAGs, none of the *E. coli* isolates qualified as one of the known major pathotypes of *E. coli* [22, 23] suggesting their commensal status. Nevertheless, they constitute potential health risk of reservoirs of VAGs. Furthermore, the pathogenic potential of some of these isolates could not be completely ruled out; particularly those which were found to harbour at least one adhesion factor and an important VAG. Those potentially pathogenic *E. coli* strains included the ones harbouring long polar fimbriae (*lpfA*) gene which is a potential virulence marker in *E. coli* [36]. Interestingly many of the isolates encoded the *lpfA* in addition to at least three other VAGs: 14.9% (egrets) and 7.6% (cattle). Furthermore, the pathogenic potential of the isolates encoding several of the other VAGs cannot be ruled out; for example, the two egret isolates (LEK 14 and LEK 17) encoded six VAGs and the one (LEK 79) encoded five VAGs. The detection of some of the VAGs in certain animal source *E. coli* isolates probably indicates the pathogenic potential or animal host marker [37].

The findings of this study showed high diversity of the *E. coli* isolates regarding to their serotype, virulence- and AMR-genotype. However, one egret and two cattle source *E. coli* isolates had the same O:H serotype and similar VAG and antimicrobial resistance genotype suggesting clonal dissemination between the animals thus calling for control measures. There was a predominance of O9 antigen in the egret and cattle source *E. coli* isolates, in contrast to prevalence of O111 in dairy cattle in Egypt [15].

There was a high prevalence of class 1 integrons compared to the rare to low prevalence of class 2 integrons in isolates of all animal species which is similar to reports of earlier studies in dairy cattle and asymptomatic humans [15, 38]. However, the prevalence of the class 1 integrons (42/54) in cattle is much higher in this study than 28 out of 114 previously reported in dairy cattle Egypt [15]. Notably also, the prevalence of class 1 integrons in *E. coli* strains of each animal in this study is more than twice the 31% earlier reported for *E. coli* strains in asymptomatic humans in Nigeria [38] thus suggesting animals as more important reservoirs of integrons in the country. Integrons play an important role in the development of multidrug resistance and dissemination of antimicrobial resistance in bacteria [39].

## Conclusion

Cattle egrets *(Bubulcus ibis),* whistling ducks *(Dendrocygna viduata*) and cattle constitute important reservoirs of highly diverse extended spectrum β-lactamase producing *E. coli* with potentials of inter-species transmission, widespread and virulence. The possible public health risks include contamination of the environment by the faeces of the migratory wild birds and cattle. The antimicrobial resistant bacteria could also spread through the food chain if beef meat is contaminated during slaughtering and butchering of cattle; through use of livestock faeces as manure; additionally, the birds can contaminate farm lands and agricultural products and water bodies with their faecal droppings. The findings of this study indicate the need for implementation of recommended guidelines for co-management of public health and habitat conservation; necessary regional specific guidelines can also be researched. Furthermore, meat hygiene should be adequately enforced at abattoirs to prevent contamination of meat; biosecurity measures should also be put in place to prevent wild bird intrusion into meat processing areas. There is need for formulation and enforcement of policies to regulate use of antimicrobials in the country; antimicrobial surveillance programme is also necessary. Public health education about health implications of indiscriminate use of antimicrobials is important.

## Methods

### Study sites

Samples were collected between January and July, 2016. Wild bird samples were taken on the banks of the river Awba and from cattle at the Bodija abattoir in Ibadan, Nigeria.

The River Awba is a second order river catchment that drains University of Ibadan, Ibadan, Nigeria. The river basin is situated between latitudes 7 ^o^ 25′58″ and 7 ^o^26’42″ and longitudes 3^o^ 53′21″ and 3^o^ 54′26″ East of Greenwich Meridian. The drainage area is 2.08 km^2^, its drainage density is 1.93 km/km^2^ [40]. The river drains some residential areas (staff quarters and student hostels), department of fisheries and aquaculture and academic departments in the Faculties of Science and Technology; after which a dam, called Awba Dam, is located. The dam supplements raw water supply to the University water treatment plant which processes and supplies water for domestic use and drinking of the University community. Fishing activity and fish breeding also take place in the dam. The trees on one of the dam banks serve as roosting sites for cattle egrets (*Bubulcus ibis*) during every dry season (early October to mid-May). The banks are also colonised by White-faced whistling ducks (*Dendrocygna viduata*) during the same period.

The cattle egrets sleep on treetops overnight. During dry season, each morning (around 7 a.m.), the birds disperse in groups in different directions into the city to look for food, and they start to return just before it gets dark (around 6.30 p.m.). When they arrive in the evening, they first land on the banks to drink water and then fly to perch on top of the trees to sleep till next morning; this is the daily routine of the egrets until the end of the dry season when they finally migrate away. Under the trees, the ground is usually littered with the faeces of the egrets.

Whistling ducks do not perch on the trees but swim in the Awba reservoir and restrict themselves only to the bank without trees when not swimming; the banks are littered with the faeces of the whistling ducks. Bodija abattoir is the main abattoir in Ibadan where cattle and other animals apart from poultry are slaughtered for meat consumption.

### Collection of faecal samples of birds and cattle

The Awba Dam was visited four times between January and March in 2016 (at intervals of 3 weeks) and in this period pooled faecal samples of egrets (*n* = 28) and whistling ducks (*n* = 24) were collected separately. To collect faecal samples of egrets, sterile aluminium foil was randomly spread under the trees in the evening and left it overnight to collect faecal droppings. Early in the morning at the next day, fresh faecal samples were collected into a sterile stool bottle. Faecal samples of ducks were randomly collected from the bank of the Awba Dam. Colour and consistency of faeces were used to discriminate between wild duck and egret samples to avoid a mixture of both. After collection, five randomly collected samples for each bird species were pooled to one sample. All faecal bird samples were transported to the microbiology laboratory within 2 h.

Cattle faecal samples (*n* = 250) were collected between April and July 2016 at the slaughter of the Bodija Abattoir. One sample per cattle was collected by the veterinarians on duty directly from the rectum into a sterile stool bottle using sterile gloves. The faecal samples were transported to the laboratory within 2 h.

### Culturing of faecal samples

Each faecal sample was thoroughly mixed inside the bottle using a sterile cotton swab stick and streaked onto 8 μg/mL cefotaxime-eosin-methylene blue agar (CEMB) (Lab M, Lancashire, UK). Afterwards, the plates were incubated aerobically at 37 °C for 18 h. At least two bacterial colonies were subcultured (depending on the number of colonies on the primary plate) on fresh plates of CEMB agar and incubated as above. Colonies were then inoculated onto a nutrient agar slope (Lab M, Lancashire, UK) and incubated as above. All 149 isolates were subjected to biochemical tests [41]. *E. coli* was identified by Gram staining, oxidase and citrate assay (negative) and indole, glucose, lactose assays (positive). *E. coli* isolates were further analysed genotypically by a microarray-based assay using the *E. coli* PanType AS-2 Kit [Abbott (Alere Technologies GmbH), Jena, Germany].

### Antimicrobial susceptibility testing

Susceptibility tests of all 149 bacterial isolates yielded 144 ones that where resistant to cefotaxime. These 144 isolates were further tested for susceptibility to other antimicrobials using disk diffusion assay on Müller-Hinton agar (Merck, Hamburg, Germany) following the guidelines of Clinical and Laboratory Standards Institute using *E. coli* ATCC 25922 as a quality control strain [42]. Nine different antimicrobial disks (Oxoid, Basingstoke, Hampshire, United Kingdom) were tested; ampicillin (10 μg), cefotaxime (30 μg), chloramphenicol (30 μg), ciprofloxacin (5 μg), gentamicin (10 μg), streptomycin (10 μg), imipenem (10 μg), ertapenem (10 μg) and tetracycline (30 μg). Diameters of zones of inhibition were measured with a ruler and interpreted according to the CLSI guidelines [43]. Only cefotaxime resistant isolates were subjected to further analysis. ESBL production was confirmed by the double disc synergy test as described by the Clinical and Laboratory Standards Institute.

### Genomic DNA extraction

Genomic DNA from the bacterial isolates was extracted using the DNeasy Blood and Tissue kit (Qiagen GmbH, Hilden, Germany) following the manufacturer’s instructions. When necessary, DNA was concentrated to at least 100 ng/μL using a SpeedVac centrifuge (Eppendorf, Hamburg, Germany) at room temperature with 1400 rpm for 30 min. Aliquots of 5 μL of genomic DNA were used directly for biotin-labelling and subsequent hybridization.

### GenoSeroTyping and antimicrobial resistance genotyping

The genoserotype of *E. coli* isolates was determined using the SeroGenoTyping AS-1 Kit (Abbott, Jena, Germany); while the AMR genotype was detected by both the CarbDetect AS-2 (for non-*E. coli* isolates) and the *E. coli* PanType AS-2 Kit. All kits were used according to manufacturer’s instructions. The Result Collector 2.0 (Abbott, Jena, Germany) automatically summarized the results.

An antibiotic resistance genotype was defined as a group of known genes conferring resistance to a family of antibiotics (e.g.*,* the genotype “*bla*CTX-M1/15” confers resistance to 3GC) (Fig. 1).

### Multiplex Labelling, hybridization, and data analysis

Extracted DNA was labelled by primer extension amplification using *E. coli* SeroGenoTyping AS-1 [44], CarbDetect AS-2 [45] or *E. coli* PanType AS-2 [46] kits according to manufacturer’s instructions. The multiplex labelling, hybridization and data analysis were described in very detail by Braun et al. 2014 [47].

## Data Availability

The complete raw data sets used during the current study are available from the corresponding author on reasonable request.

## Abbreviations

ESBLs: Extended-spectrum beta-lactamases
AMR: Antimicrobial resistance
3GC: Third generation cephalosporins
VAGs: Virulence associated genes
CEMB: Cefotaxime-eosine methylene blue agar
CREC: Cefotaxime resistant *E. coli*
MDR: Multidrug resistant
